# Prevalence, Attitudes, and Factors Motivating Conscientious Objection toward Reproductive Health among Medical Students

**DOI:** 10.1055/s-0038-1673367

**Published:** 2018-10

**Authors:** Omar Ismail Santos Pereira Darzé, Ubirajara Barroso Júnior

**Affiliations:** 1 Escola Bahiana de Medicina e Saúde Pública, Salvador, BA, Brazil

**Keywords:** legal abortion, adolescence, reproductive rights, medical education, women's health, aborto legal, adolescência, direito reprodutivo, educação médica, saúde da mulher

## Abstract

**Objective** We have evaluated the prevalence of and the motivating factors behind the refusal to provide reproductive health services and the ethical knowledge of the subject among medical students from the Escola Bahiana de Medicina e Saúde Pública, in the state of Bahia, Brazil.

**Methods** The present cross-sectional study involved 120 medical students. A questionnaire was utilized. The dependent variables were students' objections (or not) regarding three clinical reproductive health cases: abortion provided by law, contraceptive guidance to an adolescent without parental consent, and prescription of emergency contraception. The independent variables were age, gender, religion, ethical value, degree of religiosity, and attendance at worship services. Ethical knowledge comprised an obligation to state the reasons for the objection, report possible alternatives, and referral to another professional. Data were analyzed with χ^2^ tests and *t*-tests with a significance level of 5%.

**Results** Abortion, contraception to adolescents, and emergency contraception were refused by 35.8%, 17.5%, and 5.8% of the students, respectively. High religiosity (*p* < 0.001) and higher attendance at worship services (*p* = 0.034) were predictors of refusing abortion. Refusal to provide contraception to adolescents was significantly higher among women than men (*p* = 0.037). Furthermore, 25% would not explain the reason for the refusal, 15% would not describe all the procedures used, and 25% would not refer the patient to another professional.

**Conclusion** Abortion provided by law was the most objectionable situation. The motivating factors for this refusal were high commitment and religiosity. A reasonable portion of the students did not demonstrate ethical knowledge about the subject.

## Abstract (pt)

**Resumo:**

**Objetivo** Avaliar a prevalência e os fatores motivadores da recusa em prestar serviços de saúde reprodutiva, bem como o conhecimento ético do tema, entre estudantes de medicina.

**Métodos** Estudo transversal, envolvendo 120 estudantes de medicina. Aplicou-se um questionário cujas variáveis dependentes foram a existência ou não de objeções quanto à condução de três casos clínicos sobre saúde reprodutiva: o abortamento previsto em lei, a orientação contraceptiva a uma adolescente sem consentimento dos pais, e a prescrição de contracepção de emergência. As varáveis independentes foram: idade, gênero, religião, valor ético, grau de religiosidade e frequência a cultos religiosos. Os conhecimentos éticos pesquisados foram a obrigação de expor os motivos da objeção, relatar as alternativas possíveis e encaminhar a paciente a outro profissional. Os dados foram analisados pelo teste do χ2 e pelo teste-*t*, com nível de significância de 5%.

**Resultados** O abortamento foi recusado por 35,8% dos estudantes, a contracepção aos adolescentes por 17,5%, e a contracepção de emergência por 5,8%. A alta religiosidade (*p* < 0,001) e uma maior frequência a cultos (*p* = 0,034) foram os preditores identificados no caso do abortamento previsto em lei. A recusa da contracepção aos adolescentes foi significativamente maior entre as mulheres (*p* = 0,037). Entre os estudantes, 25% não explicariam o motivo da recusa, 15% não descreveriam todos os procedimentos e 25% não fariam o encaminhamento da paciente para outro profissional.

**Conclusão** O abortamento previsto em lei, foi a situação mais objetada. Os fatores motivadores a esta recusa foram o alto comprometimento e maior religiosidade. Uma parcela razoável dos alunos não demonstrou ter conhecimentos éticos sobre o tema.

## Introduction

Many diagnostic and therapeutic procedures have been refused by physicians because they are incompatible with their religious, moral, or ethical beliefs.^1^ Reproductive medicine is notably the most affected by these refusals, especially regarding termination of pregnancy, contraception, tracing of congenital malformations, and assisted reproduction techniques.^2^ ^3^ The refusal to provide these procedures impacts negatively the health of those who need them and the health system as a whole.^1^ The refusal also contradicts international human rights policies that guarantee access to high-quality reproductive and sexual health care.^4^ ^5^ End-of-life palliative care, stem cell treatment, and care related to the use of licit and illicit drugs have also been grounds for refusal by medical professionals.^6^ ^7^

Conscientious objection is exceptional and is based on ethical, moral, and religious reasons, which, in turn, must be sincere and authentic.^8^ It must have a constant character, and should not be a temporary whim, that is, a changeable position according to each circumstance.^9^ The right to freedom of religion, of conscience, and of thought is guaranteed by the International Covenant on Civil and Political Rights, considered the legal pillar of the Universal Declaration of Human Rights. However, these rights are limited to protect the well-being of others.^10^ Conscientious objection is governed by subjective considerations and certain criteria are required in order for the refusal to be legally compliant with them. The justification must be supported by reasons of an intimate, moral, or religious forum, and the requested procedure should not be an emergency; therefore, referral to other physicians to conduct the procedure is possible, without causing damage to the patient.^11^ ^12^

This topic requires further discussion and research that will contribute to a greater number of publications, thus broadening the debate among diverse professionals and health services. The available literature shows that medical professionals are unaware of the concept of “objector” and that they are obliged to justify the reason for refusal, to provide information on treatments that they consider objectionable, and to refer them to professionals who perform these procedures.^13^ Data on the prevalence of objectors are few and restricted to sites that require the physician to register as such. For example, the Italian Ministry of Health reports that 70% of the gynecologists and obstetricians and 50% of the anesthesiologists are registered as objectors to pregnancy termination.^14^ In Portugal, approximately 80% of the gynecologists refuse to perform abortions.^15^ ^16^ However, few studies have attempted to evaluate the subject in medical training.

Consequently, we have identified the prevalence of refusal to provide reproductive health services among medical students who were concluding their medical internship in perinatology. Specifically, we have examined three situations: abortion provided by law, contraceptive counseling for young adolescents, and prescribing emergency contraception (the “morning-after pill”). In addition, we have identified the motivating factors behind the objection of the students and sought to determine their ethical knowledge about the subject.

## Methods

This was a cross-sectional study that was developed between June 2016 and July 2017. The participants were 120 medical students from the Escola Bahiana de Medicina e Saúde Pública, in the state of Bahia, Brazil. The students were enrolled in the 9th semester and were included consecutively (that is, in consecutive order as they finished an internship in gynecology and obstetrics). All of them were invited to participate in the project. Those who agreed, after signing a free and informed consent form, answered an unidentified self-administered questionnaire. Those who refused to participate in the project, for any reason, were excluded.

The following independent variables were considered: age, ethnicity (self-declared), religious beliefs, gender, attendance at worship services, degree of religiosity, the ethical value in the lives of the students, and their knowledge about reproductive health. Religiosity was classified according to how it influenced and promoted meaning in the life of the interviewee, based on the agreement or disagreement with two statements: “I strive to maintain and follow my religious beliefs in all aspects of my life” and “my way of living is driven by my religion.” Both claims were derived from the Hoge scale of intrinsic religious motivation and have been extensively validated in several studies.^17^ Intrinsic religiosity was then classified as low if the students disagreed with both statements; moderate if they agreed with only one; and high if they agreed with both. Ethical value was evaluated with the agreement or not with the affirmation: “No matter what I believe in, I would rather lead an ethical life.”

The dependent variables studied were whether the students objected to the conduct of three clinical cases about reproductive health: providing an abortion to a young woman who became pregnant from an act of sexual violence, providing contraceptive guidance to a 14-year-old adolescent without parental consent, and the prescription of emergency contraception to a young woman after she had unprotected intercourse. The ethical knowledge researched was the obligation imposed on the physician to clearly state the reason for the objection; their professional duty to report all possible treatment alternatives to the patient, including those that they are morally or religiously opposed to; and the need to refer the patient to another doctor who does not object to the indicated procedure.

Statistical analyses were performed using SPSS Statistics for Windows Version 14.0 (SPSS Inc. Chicago, IL, USA) and statistical significance was set at *p* < 0.05. Word and Excel 2016 for Windows (Microsoft Corporation, Redmond, WA, USA) were used for word processing and table creation. Data were analyzed using frequency and percentage distribution; χ2 tests and *t-*tests were used for the analyses.

The present study was submitted to and approved by the Research Ethics Committee of the Escola Bahiana de Medicina e Saúde Pública, with Certificate of Presentation for Ethical Assessment (no. 54585216.6.0000.5544).

## Results

All 120 students who completed the internship in perinatology participated in the project. The general characteristics of the participants are shown in Table 1.

**Table 1:** Students' characteristics

Feature	n/N (%)
Age	24.35 ± 2.72 years
Female	90/120 (75)
Caucasian	74/120 (61.7)
Followed some religion	96/120 (80.0)
Attendance at worship services	
Never	59/120 (49.2)
Once per month	37/120 (30.8)
≥ Twice a month	24/120 (20.0)
Hoge Scale	
Low religiosity	62/120 (51.7)
Moderate religiosity	37/120 (30.8)
High religiosity	21/120 (17.5)
No matter what I believe in, I would rather lead an ethical life	77/120 (64.2)
Object to abortion provided by law	43/120 (35.8)
Object to contraceptive counseling for young adolescents	21/120 (17.5)
Object to prescribing emergency contraception	7/120 (5.8)

n, number of students; N, total sample size.

Among the students who objected to abortion provided by law, a significant association was observed with a higher attendance at worship services and with a high religious motivation as measured by the Hoge scale. The refusal to provide contraceptive guidance to young adolescents was significantly more frequent among women than men. None of the features studied was associated with the objection to prescribe emergency contraception (Table 2).

**Table 2:** Predictors of objection in the discussed clinical cases

Predictor	Object to legal abortion n/N (%) *p*-value	Object to contraceptive guidance for young adolescents n/N (%) *p*-value	Object to prescribing emergency contraception n/N (%) *p*-value
**Age**	24.9 ± 3.49 *p* = 0.075^§^	24.14 ± 3.42 *p* = 0.750^§^	24.86 ± 1.57 *p* = 0.617^§^
**Followed some religion**	37/43 (86.0) *p* = 0.216+	17/21 (81.0) *p* = 0.904+	5/7 (71.4) *p* = 0.559
**Caucasian**	27/43 (62.8) *p* = 0.850+	14/21 (66.7) *p* = 0.604+	5/7 (71.4) *p* = 0.584+
**Female**	35/43 (81.4) *p* = 0.227+	12/21 (57.1) *p* = 0.037+	7/7 (100) *p* = 0.115+
**Attendance at worship services**			
Never Once per month ≥ Twice a month	17/43 (39.5) 12/43 (27.9) 14/43 (32.6) *p* = 0.034+	11/21 (52.4) 6/21 (28.6) 4/21 (19.0) *p* = 0.984+	2/7 (28.6) 3/7 (42.9) 2/7 (28.6) *p* = 0.532+
**Hoge intrinsic religious motivation scale**			
Low Moderate High	14/43 (32.6) 14/43 (32.6) 15/43 (34.9) *p* < 0.001+	11/21 (52.4) 6/21 (28.6) 4/21 (19.0) *p* = 0.961+	2/7 (28.6) 4/7 (57.1) 1/7 (14.3) *p* = 0.289+
**Ethical value**	29/43 (67.4) *p* = 0.576+	14/21 (66.7) *p* = 0.793+	3/7 (42.9) *p* = 0.226+

Abbreviations: n, number of students; N, total sample size.

+ χ^2^-test, ^§^ *t*-test.

A percentage of 24.2% of the students did not agree that it would be ethical for the physician to clearly describe the reason for the refusal. The assertion that the professional has a duty to present all possible alternatives to the treatment in question that are supported by law, even those that he is opposed to, was not recognized by 15% of the sample. When questioned about the obligation of referral to a professional that is not opposed to the treatment, and in the impossibility that the objection would not be complied with, it was rejected by 25.8% of the students (Table 3).

**Table 3:** Ethical knowledge of students about conscientious objection

Ethical knowledge	Yes n/N (%)	No n/N (%)
Would it be ethical for the physician to clearly describe to the patient why they oppose the requested procedure?	91/120 (75.8)	19/120 (24.2)
Does the doctor have an obligation to present all possible treatment options for the patient, including those that may hurt their beliefs?	112/120 (85.0)	18/120 (15.0)
Does the objecting physician have an obligation to refer the patient to someone who does not object to the requested procedure and in the impossibility that the objection will it not be complied with?	89/120 (74.2)	31/120 (25.8)

Abbreviations: n, number of students; N, total sample size.

## Discussion

There are few data on the prevalence of conscientious objection and studies that attempt to identify the motivations for these objections. The existing reports describe generalized episodes, and there is no global mapping. In the present study, the objection of the students was identified in three clinical situations, being more prevalent in the legal abortion request, which was rejected by slightly more than one third 35.8% of the sample.

Consistently, abortion is the procedure that most arouses conscientious objection globally.^6^ In a study involving 1,174 medical students from three medical schools in the state of Piauí, Brazil, the termination of pregnancy related to sexual violence was rejected by half of them.^18^ In addition, half of the medical students who participated in a project that sought to evaluate their attitudes and opinions about abortion in Brazil were uncomfortable dealing with the interruption of pregnancy, even when they had legal protection.^19^ In the UK, almost one third 31% of a random sample of gynecology and obstetrics students labeled themselves as objectors to abortion.^20^ In Spain, a study with medical/nursing students revealed that half of them supported access to termination of pregnancy and that they would be willing to provide this service.^21^ A study conducted in South Africa with 1,308 medical students showed that one-fifth would not conduct an abortion under any circumstances. This same study reported a lower likelihood of refusal among students who were older, sexually active, further ahead in the degree, and without religious affiliation compared with their peers.^22^

Following a religion seems to be strongly associated with attitudes related to abortion.^23^ The present study demonstrated that just having a religious affiliation does not indicate a greater possibility of refusal to terminate pregnancy under the law. Refusing legal abortion was significantly more prevalent among those with high (versus low) religious motivation and with a higher attendance (versus lower) at worship services, reflecting the sincerity of the beliefs of the objector. Other characteristics such as age, gender, and moral value were not shown to be motivating objections in the studied group.

The debate about conscientious objection surfaced throughout the world with the refusal to prescribe “the morning-after pill” by some doctors and pharmacists.^24^ This objection was mainly due to the existence of a probable abortive effect described in the first publications on the subject. This hypothesis has not been confirmed by the most current studies, which have shown that the delay of the ovulation is its true mechanism of action.^25^ In southern Italy, ∼ 80% of the doctors and nurses considered themselves as objectors of emergency contraception, and Italy was the last country to release the use and marketing of the method, which only occurred after 2016.^26^ In England, 10% of the doctors consider themselves to be objectors of emergency contraception.^26^ The low prevalence of refusal to use this contraceptive method in our study may reflect the most current knowledge about the subject, mainly regarding its mechanism of action and its safety, eliminating prejudices and unjustifiable fears, which are considered predictors of its refusal.^27^ None of the characteristics evaluated in the present study were associated with a greater probability of objection to this contraceptive method, including religiosity, which is indicated by the literature as the main motivator behind the refusal to provide it.^24^

More than half of the physicians who participated in a study on teenage contraception in the United States declined to offer these services to this age group, without parental consent, even though there was conflicting jurisprudence.^24^ Contraceptive counseling to adolescents was objected to by 17.4% of the students in our sample. A greater association with the objection in providing this type of assistance was observed among women, suggesting prejudiced attitudes that are reflective of a repressive sexual education. Sexual activity among adolescents raises much discussion, especially the ethical, moral, and religious aspects that represent obstacles to safe and responsible sex for young people. Another factor motivating the refusal of contraceptive guidance to young people is the lack of knowledge by the professionals of the existing legislation on the subject. The doctor-patient relationship is governed mainly by privacy, confidentiality, secrecy, and autonomy, which are also extended to adolescents. Contraceptive counseling for girls aged 12 to 14 years, when offered carefully, does not constitute an offense, if the possibility of sexual abuse or violence is eliminated.^28^

The difficulty of access to procedures, such as those discussed, resulting from the objection of medical professionals, has a disproportionate impact on reproductive health in the poorest populations. In the regions where maternal mortality rates are high, this is related to pregnancy, childbirth, puerperium, and abortion.^4^ Catastrophic individual and population consequences have also been noted in countries with abundant resources, thus creating difficulties in the free exercise of fundamental rights.^4^ In Brazil, although conscientious objection has been regulated since 2005, access to legal abortion is hampered by the objection of physicians to perform this procedure.^29^ Objection is often not based on personal, religious, or moral reasons, but instead because the individual does not believe that the pregnancy was the result of sexual violence and demands documentation that prove that the aggression really occurred. The jurisprudence that deals with the subject is ignored, thus creating unnecessary barriers to abortion provided by law.^30^

The refusal to prescribe emergency contraception, for the undeniable damages it may cause, is considered by the Brazilian Federal Council of Medicine (CFM, in the Portuguese acronym) as an ethical infraction.^31^ In addition to being the only contraceptive method that can be used after intercourse, emergency contraception has great validity in cases of sexual violence, avoiding, on average, three out of four pregnancies.^32^ The occurrence of an unwanted pregnancy from sexual violence exposes a woman to vital risks, typically culminating in an unsafe abortion.^4^ In Brazil, young women commence sexual activity at an earlier age, which involves risks.^33^ Specifically, complications related to gestation represent the second most prevalent cause of death among adolescents aged between 15 and 19 years old.^34^ Pregnancy in this age group is often associated with situations of social vulnerability, lack of information, and difficulties in accessing specialized and quality services, especially among the neediest. The strategy advocated by the World Health Organization (WHO), with the creation of services directed to this age group, succumbed to the lack of professional preparation, denying freedom of choice and confidentiality to the adolescents, as well as promoting repressive and controlling actions that were based on moral and religious issues.^35^

The International Federation of Gynecology and Obstetrics and the WHO proposed recommendations related to the tolerance of conscientious objection in reproductive health, ruling from an ethical perspective.^4^ ^13^ This regulation is aimed to achieve a balance between the conscientious objection of the doctor and the right of the patient to have access to a quality procedure and without discrimination, recognizing how harmful the consequence of the refusal could be. Conscientious objection is a guaranteed right of the professional, as is their right of not being discriminated by their convictions. However, the tolerance of the refusal of physicians is limited by the right of the patients to obtain access to the necessary treatments, thus avoiding harm.

The obligations of the objector included clearly stating the reason for the refusal; providing information on all possible treatments, including those that they are opposed to, and refer the patient to another professional. The data we have obtained regarding the refusal of the students to complete these three duties differed from those obtained in an earlier study, which sought to assess the ethical responsibilities involved in conscientious objection to abortion provided by law among medical students, in which 54% of the academics would not refer women to another professional, and more than 70% would not provide treatment options.^18^ The influence of religion on conscientious objection and the ignorance of the legal obligations of the objector justify these attitudes. Furthermore, the difficulty of some doctors in being open with their patients about their beliefs does not allow a frank dialogue about the true reasons for the refusal. The non-referral of the patient to another professional who is not a conscientious objector is an obstacle to the performance of the requested procedure, not respecting the autonomy of the patient, disrespecting scientific integrity, and failing to fulfill the greatest duty of a physician, which is to provide quality care. These determinations respect the objector status; however, they require the physician to take responsibility for the patient until the procedure requested and supported by law is performed. The refusal should not be absolute; it should be treated as an exception, and its limits must be respected, thus preserving the dignity of all the involved.

## Conclusion

The prevalence of conscientious objection and the attitudes adopted by the sample of the present study justify that the subject should be discussed in the medical curricula, including clarifying the concept of the objector and their obligations. The deleterious effects of the refusal, especially regarding the violation of the autonomy of women and of scientific integrity, must be illuminated. In addition, promoting the knowledge of future doctors regarding the existing legislation is of fundamental importance, as this can offer students a richer experience on the subject and even modify their opinions. When invoked, conscientious objection can provoke an ethical conflict due to the confrontation between the personal values of the physician and the strong emotional shock often presented by patients seeking these services. Therefore, in these moments, effective communication skills are necessary. One of the challenges of medical education is to develop teaching/learning tools that are appropriate to the development of this competence and to collaborate with the training of professionals with a high moral and ethical standard capable of meeting the needs of the health system. Lastly, guidance on the future specialty of the students should consider potential conflicts that may arise due to their moral, ethical, or religious values concerning the performance of certain procedures.
